# Monitoring the profile of cervical cancer in a developing city

**DOI:** 10.1186/1471-2458-13-563

**Published:** 2013-06-09

**Authors:** Fábio Marques de Almeida, José Carlos de Oliveira, Edésio Martins, Maria Paula Curado, Ruffo de Freitas, Marise Amaral Rebouças Moreira

**Affiliations:** 1 Faculdade de Medicina da Universidade Federal de Goiás, Rua 235 c/ 1a avenida. s/n - S. Universitário, CEP 74605-020, Goiânia, Goiás, Brazil; 2 Population-Based Cancer Registry of Goiânia, Association Against Cancer in Goiás, Araújo Jorge Hospital, Goiânia, Brazil; 3 International Prevention Research Institute – IPRI, Lyon, France; 4 Gynecology and Breast Section, Araújo Jorge Hospital, Association Against Cancer in Goiás, Goiânia, Brazil; 5 Department of Medicine, Department of Imaging and Pathology, Federal University of Goiás, Goiânia, Brazil

**Keywords:** Neoplasias of the uterine cervix, Tendency, Incidence, Study of temporal series, Brazil

## Abstract

**Background:**

Medical records are frequently consulted to verify whether the treatment and guiding principles were correct. Determine incidence and mortality trends of *in situ* and invasive neoplasms of the uterine cervix, in the period 1988–2004 in Goiânia, Brazil.

**Methods:**

The incident cases were identified through the Population-Based Cancer Registry of Goiânia. Population data were collected from census data of the Brazilian Institute of Geography and Statistics. For mortality analysis, data were extracted from the Mortality Information System. The Poisson Regression was utilized to determine the annual incidence and mortality rates.

**Results:**

A total of 4446 cases of *in situ* and invasive neoplasms of the uterine cervix were identified. No significant reductions were verified in invasive cervical cancer rates (p = 0.386) during the study period, while *in situ* carcinomas presented an annual increasing trend of 13.08% (p < 0.001). A decreasing trend was observed for mortality (3.02%, p = 0.017).

**Conclusion:**

No reduction was observed for the incidence of invasive cancer of the uterine cervix; however, increasing trends were verified for *in situ* lesions with a consequent reduction in mortality rates. These increasing trends may be the result of recently-implemented screening programs or due to improvements in the notification system.

## Background

The implementation of cytological screenings for cervical cancer has reduced the incidence and mortality of this disease in many developed countries. However, this successful story has not been observed in developing countries. The success depends on the availability and particularly, on the quality of the screening programs implemented in each country [1]. Cervical cancer is the third most incident type of cancer in the female world population [2]. The highest incidence rates are found in Central and South America, East Africa, South and Southeast Asia, and in Melanesia [3].

The United States of America (U.S.A.) predict 12,340 new cases of cervical cancer and 4030 deaths related to cervical cancer in 2013 [4]. In Brazil, 2012 estimates predicted 17,540 new cases of cervical cancer, with an estimated risk of 19 cases per 100,000 women [2]. In Goiânia, the standard incidence rate was 33.9 in 2002, with adjusted mortality rates of 10.31 in 1984, 14.47 in 1986 and 7.95 in 2004 [5].

In 1996, the Brazilian Health Ministry launched *Viva Mulher*, the National Program for Control of Uterus and Breast Cancers. One of the objectives was to reduce the number of deaths caused by cervical cancer, either through early diagnosis via Papanicolaou test (*Pap test*) or through subsequent necessary treatments [6].

This study analyzes incidence trends of *in situ* and invasive neoplasms as well as mortality trends in neoplasms of the uterine cervix in the city of Goiânia, in the period 1988–2004.

## Methods

This is a retrospective population-based descriptive analytical study, of incident cases of neoplasms of the uterine cervix, both invasive and non-invasive, diagnosed in Goiânia, Goiás (central Brazil). The incident cases between 1988 and 2004 were obtained from the Population-Based Cancer Registry (PBCR) of Goiânia. The objective of PBCR is to estimate annual incidence and mortality rates, and to establish the distribution of the different neoplasms in the city. The PBCR of Goiânia is a database recognized by the International Association of Cancer Registries of the World Health Organization.

Population data for the city of Goiânia were obtained from the Brazilian Institute of Geography and Statistics (*in portuguese*, IBGE), from the normal censuses of 1991 and 2000 and from inter-census data for the remaining years. The Municipal Secretary of Health provided data from the Mortality Information System (MIS) for analyses regarding death. Inclusion criteria for the study included fixed residence in the city for at least one year before diagnosis date.

The analyzed data were age and extension of the lesion (*in situ* or invasive). Lesions retained by the basal membrane of the epithelium were classified as *in situ*. Microinvasive, frankly invasive, and metastatic lesions were classified as invasive. Cases that did not contain information on the extension of the tumor, but for which radiotherapy was prescribed, were classified as invasive. The diagnostic basis utilized by the PBCR were cytology, histology, clinical diagnosis, and death certificates.

After revision of the medical records, cases that presented incomplete information on the extension of the tumor, even though not used for the calculation of incidence rates, were analyzed along with all complete cases to observe influence on the final rates. Initially, these cases without information on the extension of the tumor were included with the invasive cases according to the date of diagnosis. The same cases were then included with the *in situ* cases to observe the distribution throughout the analyzed years, and to evaluate any variation in incidence rates.

Standard incidence and mortality rates were calculated using Segi’s world population, and expressed per 100,000 inhabitants. Central trend analyses were carried out to determine the average age of patients. The Poisson Regression model was applied to determine the annual incidence and increase rates, from 1988 to 2004. The model was considered to be statistically significant for values of *p* lower than 0.05 and confidence intervals of 95%.

## Results

In the period 1988–2004, 4446 cases of neoplasms of the uterine cervix were identified. Histological confirmation was available for 91.9% (4085) of the cases, while 6.4% (283) were verified by cytology, 0.5% (24) by clinical diagnosis, 1.1% (47) by death certificates and only 0.1% (7) of the cases were verified by other sources of information. The majority of the identified cases were *in situ* 49.7% (2213), while 36.1% (1603) were classified as invasive, and 14.2% (630) of the cases presented no information on the extension,

The cases with no information on the extension of the tumor (14.2%), were reclassified according to the information contained in the medical records and then distributed amongst the complete cases. This redistribution did not modify the incidence rates for *in situ* or invasive neoplasms.

**Figure 1:**
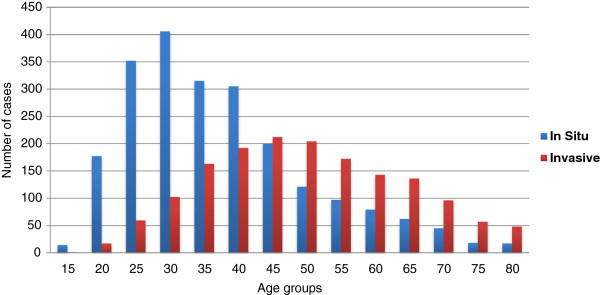
**Comparison between the incidence of *in situ *and invasive tumors distributed by age groups from 1988 to 2004 in Goiânia, Goiás, Brazil.**

The incidence rates per age group (Figure 1) show that women under the age of 45 presented a high frequency of *in situ* neoplasms while women over that threshold presented more invasive neoplasms. The average age of women presenting *in situ* neoplasms was 40 (SD ±13) while for invasive neoplasms the average age was 52 (SD ±14). Distribution by age groups for *in situ* cases showed that 32% (708) of cases occurred in the 30–49 years of age group. Regarding invasive cancer, 22.4% (359) of the patients were over 50 years old. The incidence peak for *in situ* neoplasms occurred at 30 years of age, while invasive neoplasms peaked at 45 (Figure 1).

**Figure 2:**
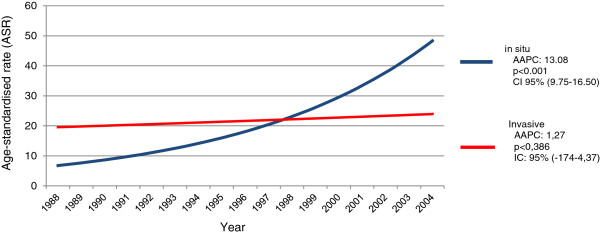
**Annual incidence trends of *in situ *and invasive cervical cancer in the period from 1988 to 2004 in Goiânia, Goiás, Brazil.** AAPC (Annual Percentage Change), CI – Confidence Interval.

The Annual Percentage Change (APC) in incidence rates of *in situ* neoplasms was 13.08% per year (*p* < 0.001) (Figure 2), while the APC for invasive tumors was 1.27% (*p* < 0.386) (Figure 2).

**Figure 3:**
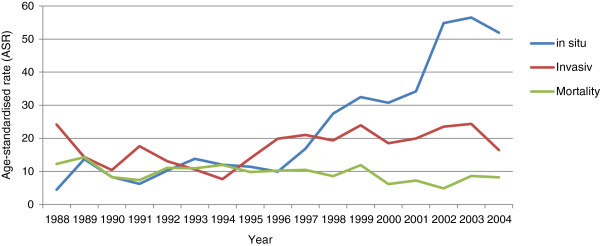
**Comparison between standard rates (per 100,000 women) of *in situ *and invasive tumors and mortality rates for cervical cancer from 1988 a 2004 in Goiânia, Goiás, Brazil.**

In 1988, standard incidence rates were 5.83 per 100,000 women for *in situ* neoplasms and 34.01 for invasive tumors; in 2004, the rates changed to 47.35 and 18.36, respectively (Figure 3).

**Figure 4:**
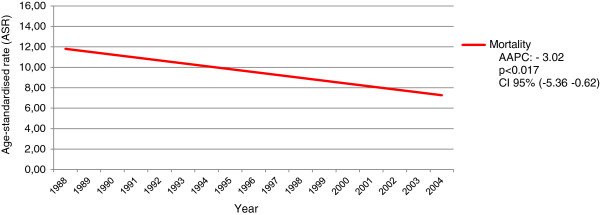
**Annual mortality trends for cervical cancer in the period from 1988 to 2004 in Goiânia, Goiás, Brazil.** AAPC (Annual Percentage Change), CI – Confidence Interval.

The highest mortality rates (14.8) of the entire study period were registered in 1989; the lowest rates (4.8) were recorded in 2002 (Figure 3). Cervical cancer mortality rates decreased 3.02% per year (*p* = 0.017) (Figure 4).

## Discussion

Epidemiological studies have demonstrated that the evolution period of a cervical lesion until its invasive form is of approximately 10 years. Such a relatively long period permits the identification of lesions in the non-invasive phase as well as it allows for effective preventive actions to be implemented through screening programs[7]. In Brazil, the model of the screening program is opportunistic; individual calling/mobilization of the target population to be screened is not practiced [8].

Throughout the entire study period (17 years), a distinct profile of increase in the incidence of *in situ* cervical neoplasms was verified in Goiânia. This observation was possible thanks to the decision of the PBCR of Goiânia to also collect information on non-invasive neoplasms, demonstrating the important role of registries in monitoring the effectiveness of screening programs over a long period of time.

*In situ* lesions, which in the first year corresponded to 15.5% of the registered cases, represented 75.7% of all cases in 2004. Such an increase may be due either to a higher level of maturity in data collection and registration or to greater coverage of the population (via cytological diagnosis). Progressive incorporation of the *Pap test* in health services has allowed for the diagnosis and treatment of precursor lesions, resulting in reduction of death rates.

The increase in the number of diagnosis of *in situ* neoplasms of the uterine cervix was higher in 1998 and thereafter. This increase approximately coincides with the launching of the program *Viva Mulher* by the Brazilian Federal government in 1996, for the prevention of cervical and breast cancers [8]. The higher number of *in situ* neoplasms diagnosed in young women indicates an increase in consciousness and self-awareness in this group of women in relation to cervical cancer. It also proves the capacity of the *Viva Mulher* program to enable an early diagnosis, resulting in a decline of mortality from cervical cancer.

The ratio between the number of cytological exams performed in women between 25 and 59 years of age and the feminine population of the same age group is utilized to indicate the coverage of the screening program in the target population. The ratio evaluates, in a direct manner, the availability of prevention and control actions against cervical cancer; ratios above 0.3 are considered adequate. In Goiânia, the ratio was 0.119 in 2004; in 2007 it almost halfed, being 0.05 [9]; obviously these ratios are below the ideal threshold. These figures indicate that the offer of *Pap tests*, and its consequent acceptance, is below the needs of the female population of the city of Goiânia.

In the city of São Paulo, southeastern Brazil, 86.1% of the women in the age group 15–59 years old had *Pap tests* in 2000 [10]. In Pelotas, in the state of Rio Grande do Sul (Southern Brazil), approximately 79% of the women between the ages of 25 and 49 affirmed to have had the test at least once in their lives [11]. This evidences the disparities present in the assistance to risk population in Brazil. There is a need to standardize the coverage of official programs to reach the female population in all states equally and to guarantee that those screened will receive effective monitoring and treatment if necessary.

The Brazilian state of Paraná, in southern of Brazil, increased the *Pap test* coverage from 43% in 1997 to approximately 86% in 2002; this was sufficient to reduce mortality by approximately 30% [12]. This also demonstrates the feasibility of implementation of an opportunistic *Pap* screening program in developing countries, which can effectively reduce cervical cancer mortality rates.

Even if screening programs presented the single objective of reducing the incidence of invasive cancers with consequent reductions in mortality, it is practically impossible to reach 100% of the population at risk with *Pap tests*. A significant proportion of women will continue to develop cervical cancer, even if the they are participating in screening programs. This occurs due to the inherent laxity in the collection of data or mistakes in diagnosis [13]. The data presented herein demonstrates that, in Goiânia, invasive cervical cancer still has not significantly reduced its incidence trends. Cervical cancer was most incident during the first years of study, from 1988 to 1998, with stable incidence even after the introduction of the *Viva Mulher* program.

In Nordic countries, screening programs are organized and exist since the 1960’s; this explains in part the reduction in the invasive disease rates and subsequent decline in deaths [14-16]. The same trend has been observed in France and Switzerland, countries that adopted programs where women voluntarily have *Pap tests* - with no obligation [17,18]. All these screening programs are effective in the control of cervical cancer, where the coverage amplitude and the satisfactory treatment and follow-up are the most important aspects of their success. The facts that women are called to have tests or submit themselves spontaneously seem to be unimportant.

In a Danish study from 1973 to 2002 with women aged between 30 and 64 years old, it was demonstrated that the organization of a screening program accelerated the decline in cervical cancer incidence rates when compared with areas where screening selection was still opportunistic [15]. Reduction in the number of deaths by cervical cancer became evident in England when women started to be periodically called in for examination [19]. This demonstrates that screening programs continue to be a key tool in the reduction of the incidence and mortality resulting from this disease.

In developing countries where screening programs are still opportunistic, many women are left out; even when they are covered by the screening program, they do not receive effective follow-ups, which masks the potential impact of these programs on the reduction of mortality [20]. Specifically, in Brazil, approximately 80% of the medical tests are still carried out following an opportunistic model [21]. In practice, due to the lack of an effective monitoring process that accounts for the natural history of the disease, the *Pap test*s are concentrated in the same female population while a significant contingent do not have access to any tests [22] - this hinders the potential impact of the screening programs.

Several international studies have demonstrated a reduction in mortality rates over the years [16,23-26], suggesting better access to diagnostic methods and to adequate and opportune treatments worldwide. Screening programs in Brazil are not based on personalized/individual calling/mobilization of the target population. For this reason, the international results do not allow for the attribution of the observed reduction in death rates to screening programs alone [23,25,26]. Studies have shown that advances in therapeutic treatments have collaborated with the decrease in mortality rates. Combination of weekly cisplatin chemotherapy with traditional radiotherapy treatment for locally advanced tumors has reduced death risk by approximately 50% since 1999, due to the reduction of local recurrence and distant metastasis [27].

Data collected herein detected a reduction of 33% in mortality rates of Goiânia between 1988 (12.2 per 100,000) and 2004 (8.1 per 100,000) (Figure 4). The rates in Goiânia are still high when compared with those of Canada (1.9/100,000), U.S.A. (1.7/100,000), Australia (1.4/100,000), and those of Finland (0.9/100,000) [3]. This indicates that the resolubility of the treatment, for early-diagnosed cases, seems to be below expectations and therefore insufficient to significantly reduce mortality rates, as has been observed in developed countries that apply organized screenings.

It should be noted that the data utilized herein was derived from death certificates. Mendonça *et al.* compared death certificates with medical records and found that 50% of the cases referred to as “cancer of the uterus in an unspecified location” actually corresponded to cervical cancer. This produced an increase of 20% in the death rates by cervical cancer [28].

Diagnosis-related difficulties could have led to the impossibility of identifying the extension of the lesion as *in situ* or invasive, which occurred for 14.2% of the cases. Similar cases might be solved through the use of more advance methods such as invasion markers by immuno-histochemics [29]. Another factor that might explain the difficulty in classifying the lesions could be related to errors of the PBCR staff during the collection period. Although the majority of these cases was registered in the beginning of the study period, they do not interfere with the final results and demonstrate a distribution similar to the complete cases.

Most diagnoses were obtained by histological confirmation, which demonstrates the quality of the collected data and the effective monitoring of the cases by PBCR, similar to that of developed countries [30].

The fact that screening in Brazil has an opportunistic characteristic allows for the inclusion of women from younger age groups and of a shorter time period between examinations. Data from São Luis, Maranhão (Northern Brazil) show that 65% of the consulted women repeated the exam in less than one year, although this group probably presented a low risk for the incidence of cervical cancer [31].

In Mexico, coverage identification was determined through population-sampling surveys: the patients themselves informed of the attendance or not to the annual tests [32]. Another measure that could be adopted by opportunistic screening programs is a modernization of the system, with centralization of data in a single database which would permit the verification of duplicity and periodicity of exams per patient and per age group of the assisted women, in private and public institutions. Professional training of those involved in all steps of the testing and follow-up processes is another action to be adopted.

## Conclusions

No reduction was observed in the incidence trends of invasive cancer of the uterine cervix; however, there has been an increasing trend in the number of *in situ* lesions with a consequent decline in mortality rates.

Although the issues associated with opportunistic screenings as well as eventual problems described in this paper do exist, the *Viva Mulher* program of the Brazilian Federal government proved to successfully reduce mortality rates of uterine cervix cancer.

The importance of monitoring the incidence of non-invasive neoplasms through RCPBs deserves to be highlighted, as it guarantees wide coverage and quality of information which will provide effective tools that address cervical cancer adequately.

## Abbreviations

AAPC: The Annual Percentage Change; ACCG: Association Against Cancer in Goiás; IBGE: Brazilian Institute of Geography and Statistics; PBCR: Population-Based Cancer Registry; MIS: Mortality Information System; CI: Confidence interval; SD: Stand Deviation.

## Competing interests

The authors have no conflict of interest to declare.

## Authors’ contributions

FMA: Literature research development of data set and write manuscript. EM and FMA: Statistical analysis and figures. JCO: Review. RFJ: Review. MPC: Concept, study desing and supervision. MARM: Concept, study desing and supervision and review. All authors read and approved the final manuscript.

## Pre-publication history

The pre-publication history for this paper can be accessed here:

http://www.biomedcentral.com/1471-2458/13/563/prepub
